# Back to the Basics: Probing the Role of Surfaces in the Experimentally Observed Morphological Evolution of ZnO

**DOI:** 10.3390/nano13060978

**Published:** 2023-03-08

**Authors:** Amanda F. Gouveia, Samantha C. S. Lemos, Edson R. Leite, Elson Longo, Juan Andrés

**Affiliations:** 1 Department of Analytical and Physical Chemistry, Jaume I University (UJI), 12071 Castelló, Spain; 2 Brazilian Nanotechnology National Laboratory (LNNano), CNPEM, Campinas 13083-970, SP, Brazil; 3 Center for the Development of Functional Materials (CDMF), Federal University of São Carlos, São Carlos 13565-905, SP, Brazil

**Keywords:** ZnO, surface energy, morphology

## Abstract

Although the physics and chemistry of materials are driven by exposed surfaces in the morphology, they are fleeting, making them inherently challenging to study experimentally. The rational design of their morphology and delivery in a synthesis process remains complex because of the numerous kinetic parameters that involve the effective shocks of atoms or clusters, which end up leading to the formation of different morphologies. Herein, we combined functional density theory calculations of the surface energies of ZnO and the Wulff construction to develop a simple computational model capable of predicting its available morphologies in an attempt to guide the search for images obtained by field-emission scanning electron microscopy (FE-SEM). The figures in this morphology map agree with the experimental FE-SEM images. The mechanism of this computational model is as follows: when the model is used, a reaction pathway is designed to find a given morphology and the ideal step height in the whole morphology map in the practical experiment. This concept article provides a practical tool to understand, at the atomic level, the routes for the morphological evolution observed in experiments as well as their correlation with changes in the properties of materials based solely on theoretical calculations. The findings presented herein not only explain the occurrence of changes during the synthesis (with targeted reaction characteristics that underpin an essential structure–function relationship) but also offer deep insights into how to enhance the efficiency of other metal-oxide-based materials via matching.

## 1. Introduction

The determination of the surface-dependent properties of materials is essential for the structure–property relationship and the rational design for their high performance. Surface properties (i.e., surface energy, atomic structures, electronic structures, etc.) make a large difference to the stability and performance of materials. Thus, it is highly desirable that these properties be tuned in order to maximize performance, since the efficiency of these systems depends on the ability to control electronic levels on surfaces and at interfaces. Nevertheless, developing an adequate design of reaction conditions for the synthesis of a desirable morphology is a complex and difficult process. In principle, it is possible to predict equilibrium morphologies once the specific surface energies of exposed crystal surfaces become available. However, to determine the precise surface structures and energies and the morphological evolution of a given material, it is necessary to carry out multiple measurements, which is a time-consuming and resource-intensive task. Quantum mechanical simulations based on first-principles calculations have been commonly used to illuminate these phenomena at a fundamental level. Combining density functional theory (DFT) with ab initio atomistic thermodynamics can be a good strategy to overcome such experimental drawbacks and allow the investigation of exposed surfaces and morphological evolution of different materials. This knowledge is also key to discovering and controlling the properties of materials with tunable multifunctionalities and more [1,2,3,4,5,6].

In addition to SnO_2_ and TiO_2_, ZnO has been investigated and extensively used in a variety of technological applications. Due to its high activity, environment-friendly nature, and low cost, it has a wide range of properties that can be applied in optics, electronics, catalysis, and gas sensing [3,7,8,9]. These multifunctional properties of ZnO are known to be strongly dependent on its morphology. However, it is a challenge to identify the surface structure, properties, and morphologies of ZnO for surface engineering [10,11,12,13,14,15].

ZnO can be synthesized into a wide range of possible morphologies, including rods, cones, bullets, cages, and hexagonal plates, depending on the synthesis method and reaction conditions used [9,16,17,18,19,20,21,22,23,24,25]. This plethora of complex morphologies appears because ZnO crystals exhibit exposed surfaces with low surface energy values and with both polar and non-polar natures. This behavior causes relative rates of crystal surface growth to be modulated by temperature, reaction time, and the presence of a surfactant, a capping agent, counterions, or a solvent in the synthesis process [23,26,27,28,29].

Surface energy is a critical descriptor of both crystal growth and morphology. It measures the energy difference per unit area between a given surface and its bulk material. Experimental measurements of this property remain challenging and have mostly been limited to extrapolations of liquid-state surface tensions [30,31]. The relative surface energy and orientation of planes in a crystal dictate its morphology, as already explained by Wulff [32]. Wulff stated that the shortest (i.e., perpendicular) distance between the center of a crystal and its surface is proportional to the energy of that surface. In 2015, we developed a methodology to obtain a set of the available morphologies (morphology map) of a given material by using the surface energy values of its exposed surfaces and the Wulff construction [33]. Since this first publication, this methodology has been used with different semiconductors, such as ZnWO_4_ [34,35], Ag_2_WO_4_ [36,37,38,39], Ag_2_O [40], MnTiO_3_ [41], CuMnO_2_ [42], Ag_2_CrO_4_ [2], TiO_2_ [43], Cu_2_O [44], and CaXO_4_ (X = Mo or W) [45], among others [46,47,48,49].

The knowledge of the surface structures and energies of ZnO is essential not only for understanding its function mechanisms [50] but also for delineating its growth mechanism during synthesis. Since the morphology of a crystal is determined by the relative magnitudes of its specific surface energies associated with different crystallographic facets, it is feasible to alter the ratios between specific surface energies to obtain crystals with morphologies other than that predicted using the Wulff construction.

How to manipulate the relative specific surface energies associated with different crystallographic planes is the central theme of the present work. In this context, the aim is to provide a new way to intelligently design the morphologies of ZnO-based materials with high robustness. This study has four challenges. The first is to obtain the available morphologies based on the Wulff construction. The second is the introduction of an innovative model to display the morphological transformations among the available morphologies by adjusting the relative surface energy values. The third consists of introducing polyhedron energy to delineate the morphological transformations along the reaction pathways in the morphology map so as to directly link surface energy variations with changes in the morphology of materials. Lastly, we will explain how the surface energy values regulate the growth process and evolution to reach a final morphology.

The paper is structured as follows. Section 2 will describe the theoretical approach used. Section 3.1 will address how the morphology map was selected to test the principles of our approach. Section 3.2 will explore the model used to investigate the effect of surface energy on the morphologies and calculate the reaction pathways from the equilibrium morphology. Section 3.3 will show how the formation energy of facet B on surface A was calculated to obtain the final morphology. Finally, the main conclusions will be summarized in the last section. The high degree of coincidence between theory and experiments makes us believe that the model might have a more general scope of application.

## 2. Theoretical Methods

The theoretical morphologies of ZnO were studied using the Wulff construction obtained through the surface energy ($E_{surf}$) values of the $\left(11\bar{2}0\right)$, $\left(10\bar{1}0\right)$, $\left(10\bar{1}1\right)$, $\left(000\bar{1}\right)$, $\left(10\bar{1}2\right)$, $\left(11\bar{2}2\right)$, and $\left(11\bar{2}1\right)$ planes reported by Na and Park [51]. The authors used the Vienna ab initio simulation package, adopting the LDA + U and PAW schemes. For the surface energy calculations, they employed periodically repeated slab geometry, which was separated by a vacuum layer of proper thickness [51]. To achieve the theoretical morphologies, the methodology proposed by our research group [33,52,53] was applied to obtain the available set of morphologies of ZnO. According to this methodology, the crystal morphology depends on the ratios between the surface energy and the crystal symmetry and structure [33,54].

The polyhedron energy ($E_{pol}$) and the percentage of contribution of each surface in the ZnO morphology are calculated. $E_{pol}$ is obtained by the following equation: $E_{pol}^{i}=\sum_{i}C_{i}\times E_{surf}^{i}$, where $C_{i}$ is the contribution of the surface area to the total surface area of the polyhedron $\left(C_{i}=A^{i}/A^{pol}\right)$ and $E_{surf}^{i}$ is the surface energy value of the corresponding surface i [37]. On the other hand, the well-known Wulff construction is a convenient method to evaluate the formation of a macroscopic surface B of orientation (*h*_2_*k*_2_*l*_2_) on a surface A of orientation (*h*_1_*k*_1_*l*_1_). The relative energy, ΔE, can be calculated by the following expression: $\Delta E=E_{surf}^{A}\left(h_{1}k_{1}l_{1}\right)cos\theta -E_{surf}^{B}\left(h_{2}k_{2}l_{2}\right)$, where $E_{surf}^{A}$ is the surface energy (per unit area) of surface A (of orientation $\left(h_{1}k_{1}l_{1}\right)$), $E_{surf}^{B}$ is the surface energy (per unit area) of surface B (of orientation $\left(h_{2}k_{2}l_{2}\right)$), θ is the angle between surfaces A and B, and the cosθ factor corresponds to the change in surface area when facets are formed [55]. According to this expression, if ΔE is negative, surface B can grow stably on surface A, i.e., the growth process takes place along the surface with lowest surface energy. This is the classical growth mechanism of Ostwald ripening, which describes the growth of smaller crystals into larger ones through diffusion in order to reduce the total surface energy [56,57,58].

## 3. Results and Discussion

### 3.1. A computational Road to Morphology

This paper provides an alternative approach for the efficient generation of the available morphologies (morphology map) of a given material, in addition to a quantitative structure–reactivity relationship based on quantum chemistry and the Wulff construction. The first step of this investigation involves the study of the bulk (unit cell).

**Figure 1:**
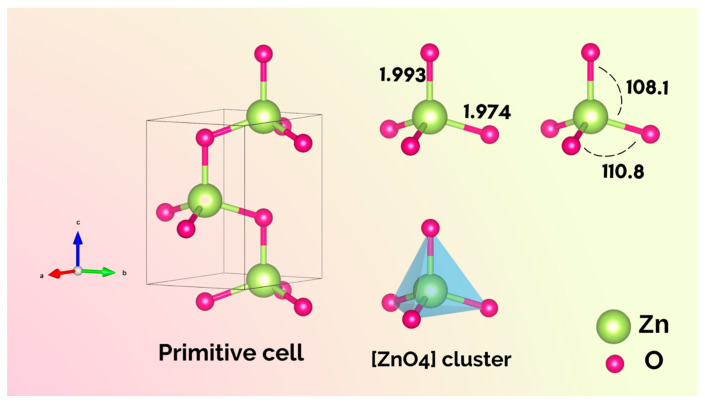
Primitive cell of the ZnO structure with [ZnO_4_] clusters, bond distances, and angles between Zn and O atoms.

The crystallographic unit cell of ZnO is shown in Figure 1. The ZnO structure is fully determined by the lattice parameters a = b and c, belongs to the space group *P*6_3_*mc*, and is formed by a two-unit formula per cell (Z = 2). In the ZnO structure, the Zn cations have a coordination number of four, which means that they are surrounded by a tetrahedron of O^2−^ anions. Therefore, the ZnO structure has [ZnO_4_] clusters as building blocks, but with some local disorder, as illustrated in Figure 1. A note on terminology: to avoid confusion, the term cluster will be used exclusively to denote the local coordination of the Zn cation corresponding to the number of neighboring oxygen anions both in the bulk and on the exposed surfaces in the morphology.

The next step consists of the investigation of surfaces that can be cut through the bulk. Previous studies have applied several different functionals to calculate the surface energy values of ZnO, as shown in Table 1.

**Figure 2:**
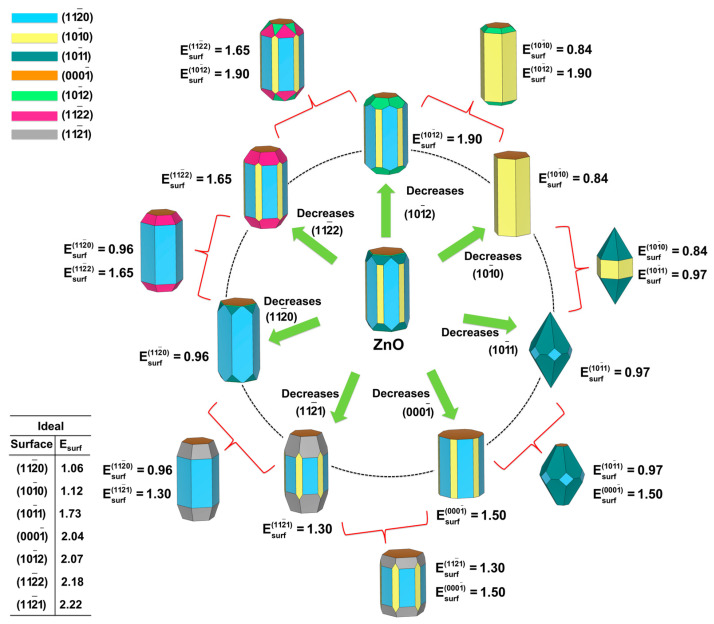
Map of available morphologies of the wurtzite ZnO.

By applying our methodology [33,52,53] and combining the surface energy values reported by Na and Park [51] and the Wulff construction, we were able to obtain a map of the available morphologies of ZnO, as illustrated in Figure 2. In the center of this figure, it is possible to see the starting morphology using the surface energy values calculated by Na and Park [51]. From this morphology, we obtained the available morphologies by decreasing the surface energy values of one (or two) surfaces using different synthesis methods.

This map becomes a powerful tool for experimentalists during the morphological characterization of materials, since it allows matching the experimental morphologies to the theoretical ones. At this point, it is possible to note that the variation in relative surface energy values is more important than their calculation, which avoids the technical drawback resulting from the fact that different functionals provide different calculated surface energy values, as observed in Table 1.

### 3.2. Where Will This Road Take Us?

This work provides insights to better understand the underlying mechanisms of crystal growth in the synthesis process. To this end, we delineated the reaction pathways that connect the different morphologies.

**Figure 3:**
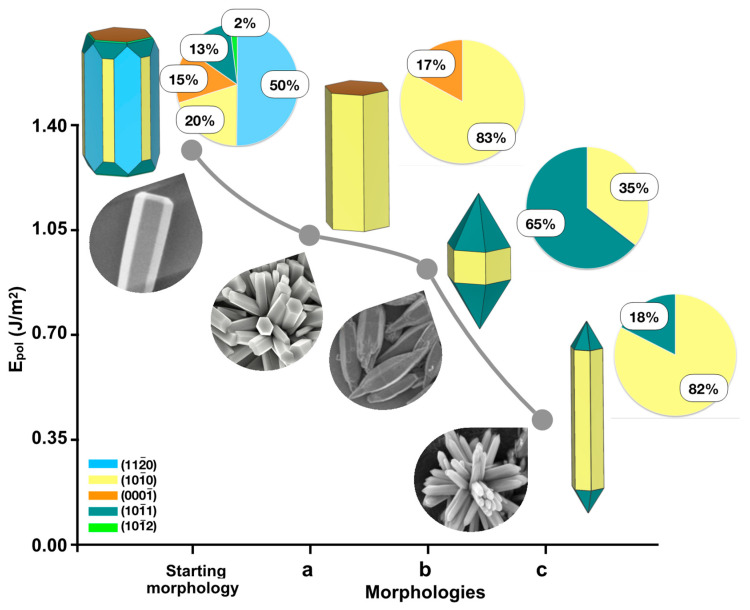
Calculated $E_{pol}$ values and the reaction pathway used in the synthesis process to obtain the most common experimental morphologies (inset): starting [9], (a) elongated hexagonal [67], (b) elongated octahedral [69], and (c) lance-shaped [70]. The percentages of each surface area are also provided for comparison purposes in a pie chart. Reprinted with permission from [9], under the terms of the Creative Commons CC—BY license. Reprinted with permission from [64], under the terms of the Creative Commons CC license. Reprinted with permission from [66]; Copyright 2020, Elsevier. Reprinted with permission from [67]; Copyright 2011, John Wiley and Sons.

Some of the morphologies displayed in Figure 2 were reported in the literature. From these morphologies, we were able to calculate the $E_{pol}$ values and build a reaction pathway for use in the synthesis process, as shown in Figure 3. It is important to note that the selected images of the morphology in Figure 3 correspond to static or steady-state values.

The starting morphology was obtained by Debroye et al. [9] and synthesized as described by Kiomarsipour and Shoja Razavi [66] by a simple hydrothermal process at low temperature without any additional surfactant, organic solvent, or catalytic agent. The elongated hexagonal morphology (a) was obtained by Amin et al. by the hydrothermal method using different experimental parameters such as pH, precursor concentration, growth time, and temperature [67]. This morphology was also reported by some of us through the doping of ZnO with Ni and Fe to enhance its photocatalytic activity [68]. These results are clear-cut examples of how the reaction conditions and the synthesis methods can modulate the final morphology.

The elongated octahedral morphology (b) was also obtained by Wu et al. by the hydrothermal method, but using water and methanol during the synthesis [69]. Zhang et al. observed a lance-shaped morphology (c) in a flower-like architecture by using different conditions in a controlled hydrothermal process (water/ethanol as a solvent and different ratio of precursors) [70].

**Figure 4:**
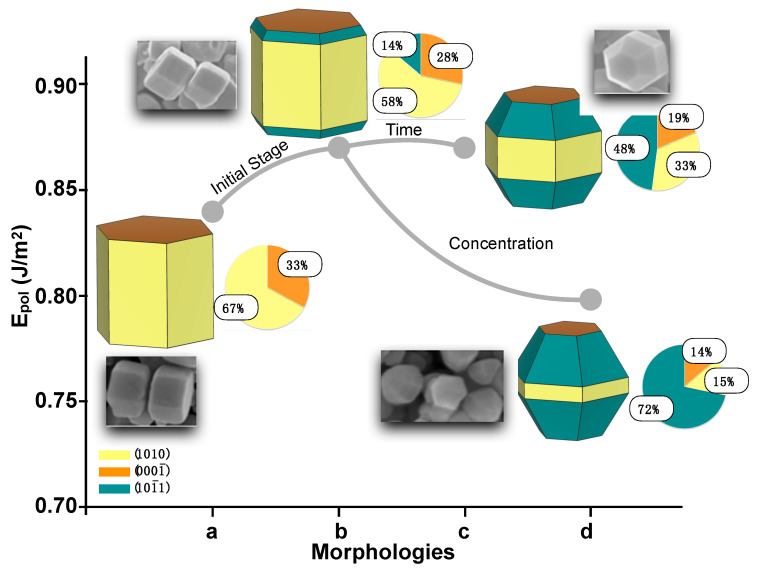
Polyhedron energy ($E_{pol}$) values in the synthesis process. The morphological evolution for the formation of different forms of tetradecahedral ZnO is highlighted. The experimental morphologies obtained by Liu et al. can be found in the inset [71]. Reprinted (adapted) with permission from [71]; Copyright 2019, American Chemical Society.

Through the solvothermal method, Liu et al. modulated the reaction conditions, i.e., reaction time and additive (tetramethylammonium hydroxide, TMAH) concentration, to obtain a wide range of morphologies of the as-synthetized ZnO samples [71]. By using the values of $E_{polyhedron}$, we were able to calculate the reaction pathway that connected the morphologies obtained by Liu et al., as illustrated in Figure 4.

As it can be seen, the morphology after the “initial stage” (b) has a higher value of $E_{pol}$ than the starting morphology (a), i.e., 0.87 J/m^2^ vs. 0.84 J/m^2^. From this point, two alternative routes can be opened as functions of synthesis process and time, resulting in two different situations to be analyzed: changes in time and TMAH concentration during synthesis. In the first case, the surface composition changes as a function of time. The contribution of the $\left(10\bar{1}0\right)$ surface decreases from 58% to 33%, whereas the contribution of $\left(10\bar{1}1\right)$ increases from 14% to 48%. However, the $E_{polyhedron}$ value does not vary, remaining 0.87 J/m^2^ (see morphology (c)). On the other hand, when the amount of TMAH is increased, the $E_{polyhedron}$ value of morphology (d) is found to be lower (0.81 J/m^2^). The contributions of $\left(10\bar{1}0\right)$ and $\left(000\bar{1}\right)$ decrease to 15% and 14%, respectively, while the contribution of $\left(10\bar{1}1\right)$ increases from 14% to 72%. This means that the stabilization of the $\left(10\bar{1}1\right)$ surface renders a more stable crystal morphology. These results can rationalize those reported by Liu et al. and explain why the $\left(10\bar{1}1\right)$ surface is more favorable for photocatalysis than the $\left(10\bar{1}0\right)$ plane [71].

### 3.3. Which Way Does the Morphology Go?

As observed, the hydrothermal method is one of the most frequently used to synthetize ZnO, with the reaction conditions being responsible for the changes in surface stability. The favorable growth directions of ZnO can be disclosed by using the Wulff construction to evaluate the formation of a macroscopic surface B of orientation (*h*_2_*k*_2_*l*_2_) on a surface A of orientation (*h*_1_*k*_1_*l*_1_).

From the surface energy values used in the construction of the morphology map, it was possible to calculate the ΔE values for all possible surfaces B on all surfaces A. These values are presented in Table 2.

By using the values of ΔE, it is possible to predict the preferential crystal growth direction of ZnO. According to the literature [10,72,73,74], the growth of ZnO crystals along the $\left[0001\right]$ direction is the most reported. Nonetheless, Cho et al. observed that when the triethyl citrate is used as a surfactant, a lateral growth of each spine along the six symmetric directions can be noted [75]. Therefore, by combining the ΔE values listed in Table 2, we could thermodynamically estimate the most favorable side surface growth along these directions, as shown in Table 3.

As it can be seen in Table 3, the combination of $\left(11\bar{2}0\right)$/$\left(10\bar{1}0\right)$, $\left(10\bar{1}0\right)$/$\left(10\bar{1}1\right)$, and $\left(11\bar{2}0\right)$/$\left(10\bar{1}1\right)$ surfaces results in a favorable crystal growth process along the [0001] direction. For the [$10\bar{1}0$] direction, the combination of $\left(11\bar{2}0\right)$/$\left(10\bar{1}1\right)$ and $\left(000\bar{1}\right)$/$\left(10\bar{1}2\right)$ also indicates a favorable crystal growth process. To further explore this behavior, it is necessary to calculate the ΔE values corresponding to the combination of surface B of orientation (*h*_2_*k*_2_*l*_2_) on surface A of orientation (*h*_1_*k*_1_*l*_1_) using the surface energy values of the morphologies depicted in Figure 3 and Figure 4. These values are presented in Table 4 and Table 5, respectively.

A detailed analysis of the results in Table 4 shows that in the starting morphology, the formation of the $\left(10\bar{1}0\right)$ plane on $\left(10\bar{1}1\right)$ is the most stable (ΔE = −2.84 J/m^2^), whereas the formation of the $\left(10\bar{1}1\right)$ surface on $\left(10\bar{1}2\right)$ is unstable (ΔE = 0.34 J/m^2^). In the case of the morphology shown in (a), in the [0001] growth direction, the formation of the $\left(10\bar{1}0\right)$ surface on $\left(000\bar{1}\right)$ is stable (ΔE = −1.75 J/m^2^), resulting in an elongated hexagonal morphology. The formation of morphologies (b) and (c) comprises only the $\left(10\bar{1}1\right)$ and $\left(10\bar{1}0\right)$ surfaces, without any growth competition, according to the ΔE values. However, by analyzing the crystal growth process in the [0001] direction, it is possible to observe that the formation of the $\left(10\bar{1}0\right)$ surface is more stable than that of $\left(10\bar{1}1\right)$ (ΔE = −2.03 J/m^2^ against ΔE = −1.04 J/m^2^, respectively; see Table 2). This explains the lance-shaped morphology depicted in (c), where the contribution of the $\left(10\bar{1}0\right)$ surface corresponds to 82% against 18% for the $\left(10\bar{1}1\right)$ plane. However, for morphology (b), these values are 35% and 65%, respectively.

According to the results presented in Table 5 for the morphologies reported by Liu et al. [71] in Figure 4, the reaction conditions change the ZnO morphologies, resulting in a stabilization of the $\left(10\bar{1}1\right)$ surface in morphology (b). Initially, the formation energy in morphology (a) stabilizes the growth of $\left(10\bar{1}0\right)$ on $\left(000\bar{1}\right)$ (ΔE = −1.22 J/m^2^). After the initial stage, as the time and additive concentration increase, the growth of $\left(10\bar{1}1\right)$ is also stabilized, as confirmed by the negative ΔE values for the combination of $\left(000\bar{1}\right)$\$\left(10\bar{1}1\right)$ surfaces. Another important fact is that the percentage contribution of the $\left(10\bar{1}0\right)$ plane decreases, whereas the contribution of $\left(10\bar{1}1\right)$ increases, as seen in Figure 4.

**Figure 5:**
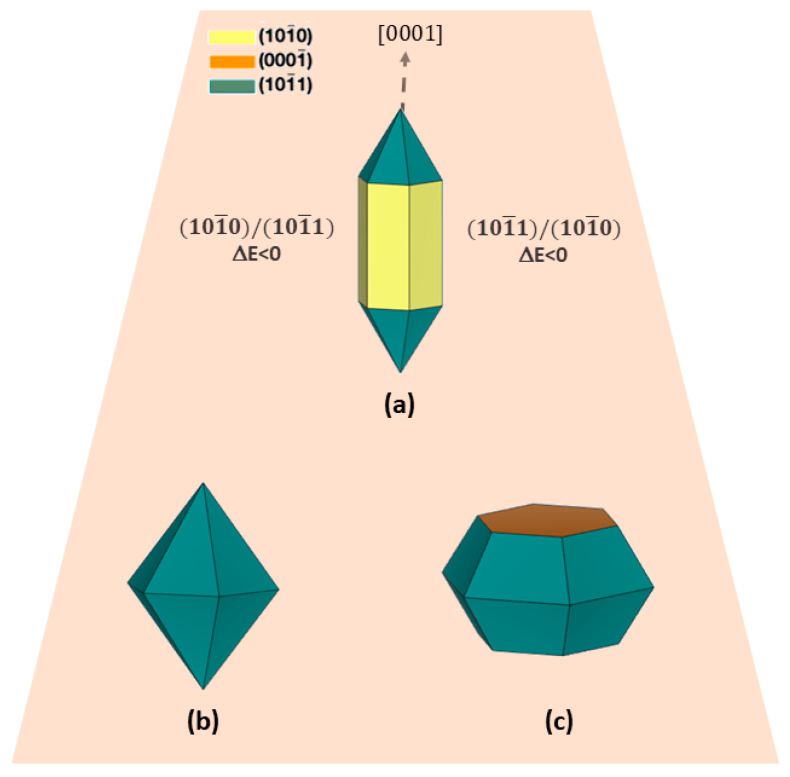
Schematic representation of (**a**) nano-bullet-like, (**b**) hexagonal cone-like, and (**c**) plate-like morphologies reported by Chang and Waclawik [20] through the combination of (0001), $\left(10\bar{1}0\right),$ and $\left(10\bar{1}1\right)$ surfaces.

Several works have used experimental characterization techniques such as XRD and TEM as valuable tools to investigate the growth direction of crystals [76,77,78,79]. For instance, Chang and Waclawik controlled morphological transformations by varying the reaction temperature and molar ratio (benzylamine/Zn^2+^ concentration from 1 to 10) [20] and obtained ZnO with a nano-bullet-like morphology exhibiting exposed $\left(10\bar{1}1\right)$ and $\left(10\bar{1}0\right)$ surfaces (Figure 5a). An analysis of the results previously reported in Table 2 shows that the combination of both surfaces provokes a thermodynamically favorable crystal growth process with ΔE < 0. As shown in Table 2, the growth in the [0001] direction is favored when these surfaces are combined, as observed in the elongated nano-bullet-like morphology (see (a) in Figure 5). By decreasing the synthesis temperature from 210 to 170 °C, a hexagonal cone-like morphology with an $\mathrm{exposed}\;\left(10\bar{1}0\right)$ surface can be obtained (see (b) in Figure 5), while an increase in the benzylamine/Zn^2+^ molar ratio results in a 2D plate-like morphology with an exposed (0001) surface (see (c) in Figure 5).

Ahmed et al. prepared ZnO nanocrystals by the hydrothermal method. Single ZnO nanorods were transformed into sharp sword-like tips by increasing the reaction time to 30 min. After 60 min of reaction, a single ZnO semi-hollow nanorod (pyramid-like morphology) was obtained [80]. This can be supported by the HRTEM images, which depict a lattice spacing corresponding to the distance between the (002) planes in the obtained ZnO structures growing along the [0001] direction. According to the results in Table 2, the growth process along the [0001] direction is favored when different surfaces are combined. In the synthesis process, the final morphology is dependent on the growth velocity, which in turn is affected by the nonuniformity and variability of the precursor solution throughout the reaction time.

**Figure 6:**
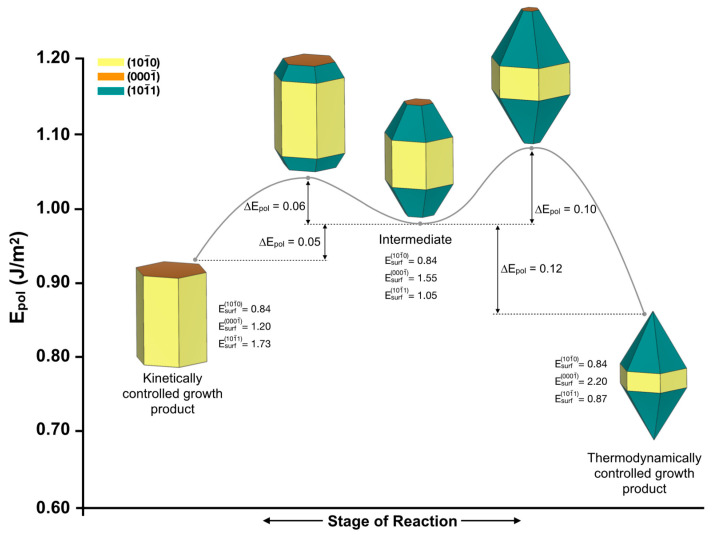
Schematic illustration of the reaction pathways connecting the kinetically and thermodynamically controlled morphologies reported by Liu and Liu [81].

Interesting reaction pathways were proposed by Liu and Liu by employing the pulsed-laser deposition technique to obtain a given morphology of ZnO [81]. The authors demonstrated that laser-induced crystal growth is a practical tool to tune the morphology of nanomaterials in a precise and effective manner. The growth directions of the ZnO crystallization in a hydrothermal reaction were selected by adjusting the laser irradiation conditions (power and time) to control the appearance of the final morphology (hexagonal versus pyramid-like, corresponding to the kinetic and thermodynamic reaction pathways, respectively). The above results can be rationalized by tuning the surface energy values of $\left(000\bar{1}\right)$, $\left(10\bar{1}0\right)$, and $\left(10\bar{1}1\right)$. From the kinetically controlled growth product with surface energy values of 1.20, 0.84, and 1.73 J/m^2^, respectively, a need arises: to overcome an energy barrier of 0.11 J/m^2^ in order to obtain an intermediate morphology. This pathway is achieved by increasing or decreasing the surface energy value of the $\left(000\bar{1}\right)$ and $\left(10\bar{1}1\right)$ surfaces to 1.55 and 1.05 J/m^2^, respectively. From this intermediate morphology, it is possible to obtain a thermodynamically controlled growth product by increasing or decreasing the surface energy values of $\left(000\bar{1}\right)$ and $\left(10\bar{1}1\right)$ to 2.20 and 0.87 J/m^2^, respectively. This results in energy barriers from the intermediate to the kinetically and thermodynamically controlled morphologies reported by Liu and Liu of 0.11 and 0.22 J/m^2^, respectively. A schematic illustration of the reaction pathways is presented in Figure 6.

## 4. Conclusions

In this study, we selected ZnO to evaluate the importance of our computational method created to reciprocate findings on morphologies and crystal growth processes from experiments. Based on the surface energy values and the Wulff construction, this strategy was found to be very useful for unraveling the morphologies that are challenging to characterize experimentally. This work provides a theoretical framework that requires as input data the surface energy values to obtain the available morphologies of a given semiconductor, its polyhedron energy, and the reaction pathways involved in a synthetic road to achieve a certain morphology. Important information can also be taken from the results presented herein for further research on the effects of morphological control on the synthesis of semiconductors. The figures of merit in this morphology map agree with the experimental images obtained by field-emission scanning electron microscopy. The high degree of coincidence between the theory and the experiments makes us believe that the model might have a more general scope of application, which, to the best of our knowledge, has not been discussed in previous literature. Additional studies are in progress to standardize this computational procedure by proving its efficiency in replicating experimental data, as well as the usefulness of techniques employed to predict structural, physical, chemical, and dynamic properties of materials. We also expect that these new insights will guide researchers aiming to apply the potential of computational methods to illustrate minute details of various types of materials.

## Figures and Tables

**Table 1:** Reported surface energy values of ZnO (in J/m^2^).

(100)	(110)	(001)	(102)	(101)	(112)	(111)	$\left(00\bar{1}\right)$	Functional	Ref.
1.12	1.06	–	2.07	1.73	2.18	2.22	2.04	LDA + U	[51]
2.05	1.16	2.00	–	–	–	–	–	B3LYP	[59,60,61]
0.87	–	–	–	2.00	–	–	1.39	PBE-D3	[3]
0.82	–	2.37	–	–	–	–	1.01	PBE	[62]
0.91	1.64	1.74		1.58				GGA	[63]
1.19	1.23							LDA	[64]
0.680 3.796	–	0.897 8.671 *	2.292 2.481 2.389	–	–	–	–	PBE	[65]

* terminal in O.

**Table 2:** Calculated values of relative energy (ΔE) of surface B on surface A (J/m^2^) and angle (θ, degree) between planes A and B.

A\B	$\left(11\bar{2}0\right)$	$\left(10\bar{1}0\right)$	$\left(000\bar{1}\right)$	$\left(10\bar{1}1\right)$	$\left(10\bar{1}2\right)$	$\left(11\bar{2}1\right)$	$\left(11\bar{2}2\right)$
$\left(11\bar{2}0\right)$	–	30.00° ΔE = − 0.96	90.00° ΔE = − 2.51	40.37° ΔE = − 2.68	53.98° ΔE = − 2.96	17.33° ΔE = − 2.16	31.97° ΔE = − 1.28
$\left(10\bar{1}0\right)$	30.00° ΔE = − 0.89	–	90.00° ΔE = − 2.54	28.39° ΔE = − 2.84	47.23° ΔE = − 3.18	34.24° ΔE = − 3.28	42.72° ΔE = − 1.84
$\left(000\bar{1}\right)$	90.00° ΔE = − 1.97	90.00° ΔE = − 2.03	–	61.61° ΔE = − 1.04	42.77° ΔE = − 1.36	72.67° ΔE = − 4.09	58.03° ΔE = − 1.99
$\left(10\bar{1}1\right)$	40.37° ΔE = − 2.60	28.39° ΔE = − 2.84	61.61° ΔE = − 1.45	–	18.84° ΔE = − 0.34	29.67° ΔE = − 2.53	26.09° ΔE = − 1.19
$\left(10\bar{1}2\right)$	53.98° ΔE = − 2.80	47.23° ΔE = − 3.18	42.77° ΔE = − 1.32	18.84° ΔE = 0.34	–	38.73° ΔE = − 1.16	27.43° ΔE = − 3.56
$\left(11\bar{2}1\right)$	17.33° ΔE = − 0.94	34.24° ΔE = − 3.23	72.77° ΔE = − 4.08	29.67° ΔE = − 2.12	38.73° ΔE = − 0.94	–	14.64° ΔE = − 3.25
$\left(11\bar{2}2\right)$	31.97° ΔE = 0.79	42.72° ΔE = − 0.45	58.03° ΔE = − 1.84	26.09° ΔE = − 0.48	27.43° ΔE = − 3.52	14.64° ΔE = − 3.27	–

**Table 3:** Signs of relative energy (ΔE) values of surfaces.

Side Surfaces	Growth Directions	
	$\left[0001\right]$	$\left[10\bar{1}0\right]$
$\left(11\bar{2}0\right)$ $\backslash \left(10\bar{1}0\right)$	ΔE<0\ΔE < 0	ΔE < 0\–
$\left(10\bar{1}0\right)$ $\backslash \left(10\bar{1}1\right)$	ΔE<0 \ΔE < 0	\ΔE < 0
$\left(11\bar{2}0\right)$ $\backslash \left(10\bar{1}1\right)$	ΔE<0\ΔE < 0	ΔE<0\ΔE < 0
$\left(10\bar{1}0\right)$ $\backslash \left(000\bar{1}\right)$	ΔE < 0\–	–ΔE < 0
$\left(000\bar{1}\right)$ $\backslash \left(10\bar{1}2\right)$	–ΔE < 0	ΔE<0\ΔE < 0

**Table 4:** Calculated values of relative energy (ΔE) of surface B on surface A (J/m^2^) and angle (θ, degree) between surfaces A and B for the crystal morphologies reported in Figure 3.

A\B			
(Starting morphology) $\left(000\bar{1}\right)$$\backslash \left(10\bar{1}2\right)$ 42.77° ΔE = − 1.36	(Starting morphology) $\left(10\bar{1}2\right)$$\backslash \left(000\bar{1}\right)$ 42.77° ΔE = − 1.32	(a) $\left(000\bar{1}\right)$$\backslash \left(10\bar{1}0\right)$ 90.00° ΔE = − 1.75	(a) $\left(10\bar{1}0\right)$$\backslash \left(000\bar{1}\right)$ 90.00° ΔE = − 2.42
$\left(10\bar{1}2\right)$$\backslash \left(10\bar{1}1\right)$ 18.84° ΔE = 0.34	$\left(10\bar{1}1\right)$$\backslash \left(10\bar{1}2\right)$ 18.84° ΔE = − 0.34	(b) $\left(10\bar{1}1\right)$$\backslash \left(10\bar{1}0\right)$ 28.39° ΔE = − 1.80	(b) $\left(10\bar{1}0\right)$$\backslash \left(10\bar{1}1\right)$ 28.39° ΔE = − 1.80
$\left(10\bar{1}1\right)$$\backslash \left(10\bar{1}0\right)$ 28.39° ΔE = − 2.84	$\left(10\bar{1}0\right)$$\backslash \left(10\bar{1}1\right)$ 28.39° ΔE = − 2.84		
$\left(10\bar{1}1\right)$$\backslash \left(11\bar{2}0\right)$ 40.37° ΔE = − 2.60	$\left(11\bar{2}0\right)$$\backslash \left(10\bar{1}1\right)$ 40.37° ΔE = − 2.68	(c) $\left(10\bar{1}1\right)$$\backslash \left(10\bar{1}0\right)$ 28.39° ΔE = − 1.26	(c) $\left(10\bar{1}0\right)$$\backslash \left(10\bar{1}1\right)$ 28.39° ΔE = − 1.27
$\left(10\bar{1}0\right)$$\backslash \left(11\bar{2}0\right)$ 30.00° ΔE = − 0.89	$\left(11\bar{2}0\right)$$\backslash \left(10\bar{1}0\right)$ 30.00° ΔE = − 0.96		

**Table 5:** Calculated values of relative energy (ΔE) of surface B on surface A (J/m^2^) and angle (θ, degree) between planes A and B for all crystal shapes reported in Figure 4.

A\B			
(a) $\left(000\bar{1}\right)$$\backslash \left(10\bar{1}0\right)$ 90.00° ΔE = −1.22	(a) $\left(10\bar{1}0\right)$$\backslash \left(000\bar{1}\right)$ 90.00° ΔE = −1.23	(c) $\left(000\bar{1}\right)$$\backslash \left(10\bar{1}1\right)$ 61.61° ΔE = −0.61	(c) $\left(10\bar{1}1\right)$$\backslash \left(000\bar{1}\right)$ 61.61° ΔE = −0.55
		$\left(10\bar{1}1\right)$$\backslash \;\left(10\bar{1}0\right)$ 28.39° ΔE = −1.73	$\left(10\bar{1}0\right)$$\backslash \left(10\bar{1}1\right)$ 28.39° ΔE = −1.73
(b) $\left(000\bar{1}\right)$$\backslash \left(10\bar{1}1\right)$ 61.61° ΔE = −0.78	(b) $\left(10\bar{1}1\right)$$\backslash \left(000\bar{1}\right)$ 61.61° ΔE = −0.49	(d) $\left(000\bar{1}\right)$$\backslash \left(10\bar{1}1\right)$ 61.61° ΔE = −0.51	(d) $\left(10\bar{1}1\right)$$\backslash \left(000\bar{1}\right)$ 61.61° ΔE = −0.58
$\left(10\bar{1}1\right)$$\backslash \left(10\bar{1}0\right)$ 28.39° ΔE = −1.90	$\left(10\bar{1}0\right)$$\backslash \left(10\bar{1}1\right)$ 28.39° ΔE = −1.90	$\left(10\bar{1}1\right)$$\backslash \left(10\bar{1}0\right)$ 28.39° ΔE = −1.63	$\left(10\bar{1}0\right)$$\backslash \left(10\bar{1}1\right)$ 28.39° ΔE = −1.63

## Data Availability

Not applicable.
